# Early‐Stage Adhesion Dynamics of Viral Hemorrhagic Septicemia Virus in Olive Flounder, *Paralichthys olivaceus*

**DOI:** 10.1111/jfd.70005

**Published:** 2025-06-25

**Authors:** Su‐Young Yoon, Yo‐Seb Jang, Soo‐Jin Kim, Myung‐Joo Oh

**Affiliations:** ^1^ Department of Aqualife Medicine Chonnam National University Yeosu Republic of Korea; ^2^ Department of Aqualife Medicine Mokpo National University Mokpo Republic of Korea

**Keywords:** olive flounder, ultra centrifugal filter, viral adsorption, viral hemorrhagic septicemia virus

## Abstract

Viral hemorrhagic septicemia virus (VHSV) is a major pathogen in olive flounder ( *Paralichthys olivaceus* ) aquaculture, leading to high mortality rates and significant economic losses. Recurrent outbreaks underscore the need for a deeper understanding of the early‐stage adhesion and infection mechanisms of VHSV. To address this gap, this study investigated the adsorption and replication dynamics of VHSV in various olive flounder tissues under different temperature conditions (15°C, 20°C, and 25°C). Viral RNA levels were quantified using RT‐PCR, and viral recovery from seawater was assessed using Centricon ultrafiltration (30 kDa). VHSV was detected in the gills and mucus within 1 h post‐infection, with peak viral loads observed between 1 and 3 h at 15°C and 20°C, indicating that these external tissues serve as initial adsorption sites. A marked increase in viral load in the mucus at 12 h post‐infection suggests that mucus not only contributes to early viral capture but may also facilitate re‐release into the environment. In tanks containing both fish and virus, viral concentrations were initially lower than those in virus‐only controls but eventually equalised, supporting the hypothesis that adsorbed viruses can be released back into the water. The ultrafiltration method demonstrated recovery efficiency, exceeding 90% at viral concentrations above 10^3^ copies/200 mL, demonstrating its effectiveness for environmental monitoring of VHSV. Overall, VHSV initially adheres to the gill and mucus layers before spreading internally, with host and environmental factors potentially influencing its persistence and transmission in aquaculture systems.

## 1 Introduction

The initial stage of viral infection involves the attachment and entry of viral particles into host cells, a process fundamental to the establishment of infection (Sobhy 2017). Viral adhesion occurs either through direct fusion with the host cell membrane or by binding to specific receptors on the cell surface, thereby facilitating viral entry and subsequent replication (Whittaker et al. 2000; Bhella 2015). Once inside the host cell, the virus replicates and produces progeny virions, which spread to adjacent tissues, further propagating the infection (Mothes et al. 2010; Kutter et al. 2018). As the attachment phase plays a critical role in determining viral infectivity, a comprehensive understanding of the mechanisms of viral adhesion is essential for the development of effective control strategies.

Viral hemorrhagic septicemia virus (VHSV) is a negative‐sense single‐stranded RNA virus classified under the genus *Novirhabdovirus* within the family *Rhabdoviridae* (Fauquet et al. 2005). This pathogen has been documented to infect over 80 species of freshwater and marine fish globally (Skall et al. 2005), with outbreaks causing significant morbidity and mortality in economically important species such as salmonids and olive flounder ( *Paralichthys olivaceus* ). These outbreaks have led to considerable economic losses in aquaculture production (Isshiki et al. 2001; Nishizawa et al. 2011). In South Korea, a VHSV outbreak in 2001 resulted in mortality rates ranging from 40% to 60% among cultured olive flounder, causing substantial financial damage to the aquaculture industry (Kim et al. 2009).

To mitigate the impact of VHSV in aquaculture, extensive efforts have been directed towards the development of effective vaccines (Nishizawa et al. 2011; Kim and Oh 2020; Jang et al. 2023). However, despite these efforts, outbreaks continue to occur, highlighting the need for a more comprehensive understanding of VHSV infection dynamics, particularly the early mechanisms of viral adhesion. Previous studies suggest that a minimum viral concentration of 10^3.7^ TCID_50_/mL is necessary to establish infection, emphasising the necessity of initial adhesion to the host's external surfaces prior to successful infiltration of target tissues (Yoon et al. 2024).

Fish viruses primarily adhere to epithelial surfaces that are directly exposed to water, including the fin bases, gills, and skin of fish (Costes et al. 2009; Krishnan et al. 2020; Løkka et al. 2023). Among these external surfaces, the gills are considered particularly important for viral colonisation due to their extensive surface area, high vascularization, and constant exposure to the aquatic environment. These anatomical and physiological characteristics provide a favourable interface for viral adhesion, accumulation, and subsequent invasion into deeper tissues (Evans et al. 2005; Lovy et al. 2007). In the case of rhabdoviruses, the gills and fin bases have been proposed as major sites of initial viral adsorption, potentially acting as primary entry points during the early stages of infection (Kim and Faisal 2011; Dixon et al. 2016).

Detecting VHSV in aquatic environments presents several challenges, primarily due to the typically low concentration of viral particles dispersed across large volumes of water. Therefore, virus concentration techniques are essential for enhancing detection sensitivity and enabling accurate quantification of viral adhesion and shedding patterns. Previous studies have employed negatively charged membrane filtration, hollow fibre filtration, and precipitation‐based methods to concentrate viruses from water samples (Jee et al. 2013; Kim et al. 2015; Ryu et al. 2023). However, these methods face limitations in processing large sample volumes or detecting low viral titers, making it challenging to accurately characterise viral adhesion on fish surfaces. To address these limitations, this study employed Centricon filtration‐based concentration techniques, which are known to enhance viral recovery and detection sensitivity while enabling the simultaneous processing of multiple water samples (Ahmed et al. 2020). Accordingly, a virus concentration method was applied to quantify VHSV adsorption and examine attachment dynamics in relation to immersion duration.

This study aimed to identify the specific sites of VHSV adhesion in olive flounder and to elucidate the dynamics of early‐stage infection. To achieve this, we initially assessed the performance of the viral concentration technique and subsequently investigated VHSV adsorption across various tissues and immersion durations to clarify the dynamics of early infection. Ultimately, this study seeks to provide foundational data to support vaccine development and aquaculture disease management, thereby contributing to the reduction of viral transmission and associated economic losses.

## 2 Materials and Methods

### 2.1 Virus and Fish

The VHSV isolate, FYeosu05 (Genotype IVa) was propagated in fathead minnow (FHM) cells cultured in 75 cm^2^ tissue culture flasks at 15°C ± 0.5°C. The cells were maintained in Dulbecco's minimum essential medium (DMEM; Gibco, USA) supplemented with 5% (v/v) fetal bovine serum (FBS; Gibco), 150 IU/mL penicillin G, and 100 μg/mL streptomycin (Gibco). After observing complete cytopathic effects (CPE), the cell culture supernatant was collected and centrifuged at 4000 rpm for 15 min at 4°C. The virus‐containing supernatant was aliquoted and stored at −80°C until further use.

All animal experiments were approved by the Chonnam National University Institutional Animal Care and Use Committee (CNU IACUC‐YS‐2024‐5). Fish were reared in isolated tanks supplied with UV‐treated seawater. Prior to the experiment, individuals were randomly selected from the tanks and tested for the absence of VHSV using reverse transcription polymerase chain reaction (RT‐PCR).

### 2.2 Evaluation of Virus Recovery Rate

The virus recovery rate was assessed by comparing the values of the diluted sample before and after the virus concentration process. Following filtration through a 0.22 μm PES filter (Biofil, China), 1 mL of VHSV (FYeosu 05 strain) was added to 200 mL of sterilised seawater and thoroughly mixed. The mixture was concentrated using an Amicon Ultra centrifugal filter unit (Amicon, Ireland, 30 kDa) until the final volume was reduced to 200 μL. Subsequently, elution was performed with 1X HBSS solution (Gibco, USA) to adjust the final volume to 1 mL. To compare the viral loads before and after the concentration process, RNA was extracted using the PureLink Viral RNA/DNA Mini Kit (Invitrogen, USA). Complementary DNA (cDNA) synthesis was performed using 5 μL of extracted RNA according to the manufacturers protocol with the ReverTra Ace RT Kit (Toyobo, Japan). Viral concentrations before and after filtration were subsequently quantified using RT‐PCR. The detailed qPCR conditions, including primer sequences and thermal cycling parameters, are provided in Section 2.5.

The virus recovery rate was calculated as follows:

$\text{Virus recovery}\;\left(\%\right)=\frac{\text{number of in eluant or concentrate}}{\text{number of virus in spiked sample}}\times 100$.

### 2.3 Adsorption Experiment

The adsorption experiment was conducted in triplicate. In the first trial, fish had a mean body weight (MBW) of 6.2 ± 1.98 g and a mean length of 9.85 ± 0.75 cm. In the second trial, fish had an MBW of 2 ± 0.05 g and a mean length of 7.17 ± 0.37 cm. In the third trial, fish had an MBW of 1.5 ± 0.2 g and a mean length of 6 ± 0.5 cm. The seawater used in all trials was pre‐filtered through a 0.22 μm PES filter (Biofil, China). The experiment was conducted using an immersion method, comparing viral loads in seawater from tanks containing fish to those in tanks without fish at time points of 1, 3, 6, and 12 h. At each time point, 200 mL of seawater was collected, and gill and fin tissues were sampled from the olive flounder to compare viral adsorption between the two sites.

### 2.4 Tissue Sampling and Viral Quantification Under Different Temperature Conditions

Olive flounder were acclimated and maintained in three separate tanks set at 15°C, 20°C, and 25°C, respectively. Fish were divided into groups and immersed in VHSV at a concentration of 10^5.5^ TCID_50_/mL. Control groups were immersed in an equivalent volume of sterile DMEM. At 0, 1, 3, 6, 12, 24, and 48 h post‐infection (hpi), fish were randomly sampled from each temperature group (*n* = 3 per time point). The following tissues were aseptically collected: mucus, blood, gill, heart, spleen, kidney, and muscle. All samples were immediately stored at −80°C until RNA extraction.

### 2.5 Quantifying Infection Using qPCR

Total RNA was extracted from tissue samples using Tri reagent according to the manufacturer's instructions. In contrast, RNA from seawater samples was extracted following the method described in Section 2.2. The concentration and purity of the extracted RNA were measured using a NanoDrop spectrophotometer (Thermo Fisher Scientific, USA). Subsequently, cDNA was synthesised from the RNA using the ReverTra Ace qPCR RT Kit (Toyobo, Japan). Real‐time PCR was performed using a CFX Connect Real‐Time PCR Detection System (Bio‐Rad, USA) with AccuPower 2X GreenStar qPCR Master Mix (Bioneer, Korea). The thermal cycling conditions consisted of an initial denaturation at 95°C for 5 min, followed by 40 cycles of 95°C for 10 s and 59°C for 30 s. All reactions were conducted in triplicate. The viral load was quantified using genotype‐specific qPCR assays, following the method previously described by Kim et al. (2014). The primer sequences used for the qPCR assays are listed in Table 1.

**TABLE 1:** qPCR Primers and genes used in the study.

Primer name	Gene	Primer sequence (5′ → 3′)	Amplicon length (bp)
VHSV G_2 F	G	AAAACCATCCTGGAGGCAAAGC	364
VHSV G_2 R	G	TTCGGGGAAGAAAGGGTACTCG	447

### 2.6 Statistical Analysis

Among the experiments conducted in this study, statistical analysis was conducted only for the tissue‐specific viral replication data. The effects of time, temperature, and their interaction were analysed using two‐way ANOVA in GraphPad Prism 9.0 (GraphPad Software Inc., San Diego, CA, USA), with *p*‐values < 0.05 considered statistically significant. All other experimental results were interpreted descriptively.

## 3 Results

### 3.1 Recovery Rate

To evaluate the efficiency of virus recovery, viral cDNA copies were quantified before and after the concentration process using RT‐PCR. The results are presented in Table 2. Recovery rates varied depending on the initial viral concentration in seawater. At lower concentrations (3.43E+02 copies/200 mL), the mean recovery rate was 63.21% ± 35.33%. As the initial viral concentration increased, recovery efficiency improved, reaching 94.01% ± 30.05% at 2.53E+04 copies/200 mL. At higher concentrations, such as 3.16E+05 and 8.75E+06 copies/200 mL, the recovery rates slightly exceeded 100%, with values of 109.83% ± 15.24% and 107.18% ± 9.45%, respectively. These results indicate that the virus concentration method using the Amicon Ultra centrifugal filter (30 kDa) effectively recovered VHSV from seawater samples.

**TABLE 2:** Recovery efficiency of VHSV from seawater at different initial concentrations.^a^

Initial concentration (viral cDNA copies/200 mL seawater)	Recovered concentration (viral cDNA copies/mL)	Mean recovery (%)
3.43E+02	1.97E+02 ± 1.21E+01	63.21 ± 35.33
3.01E+03	2.65E+03 ± 3.74E+02	87.98 ± 12.45
2.53E+04	2.38E+04 ± 7.61E+03	94.01 ± 30.05
3.16E+05	3.47E+05 ± 4.81E+04	109.83 ± 15.24
8.75E+06	9.38E+06 ± 8.27E+05	107.18 ± 9.45

^a^ Mean ± standard deviation.

### 3.2 Temporal Variations in Viral Load Across Different Water Temperatures

**FIGURE 1:**
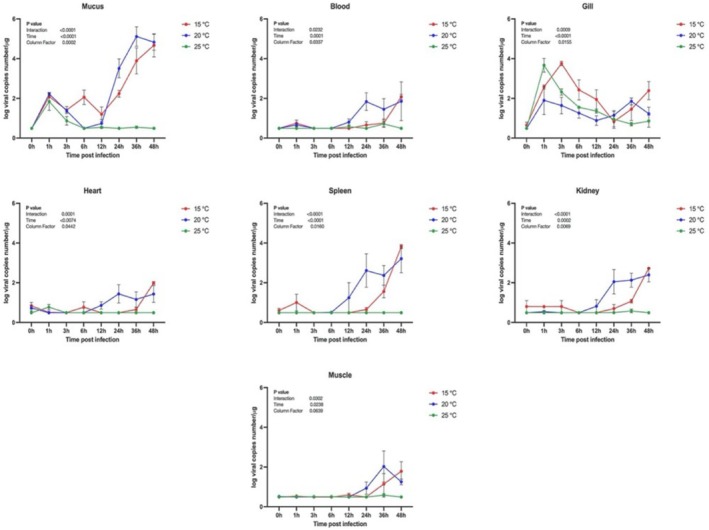
Time‐course of VHSV RNA copy numbers in mucus, blood, gill, heart, spleen, kidney, and muscle tissues of infected fish maintained at three different temperatures (15°C, 20°C, 25°C). Viral loads were quantified using qRT‐PCR and are expressed as log10 RNA copies/μg total RNA.

Viral replication in external gill and mucosal tissues exceeded 100 copies within the first hour post‐infection. Although all temperature groups exhibited viral loads exceeding 100 copies in mucus within the first hour, a rapid increase was observed thereafter, except in the 25°C group, where no further viral proliferation was observed beyond 12 h post‐infection. In the gills, the highest viral loads were recorded during the initial 1–3 h post‐infection, followed by a gradual decrease. After 24 h, viral replication had significantly reduced in all groups, except those maintained at 15°C. In internal organs and tissues such as the heart, spleen, kidneys, muscle, and blood, a consistent increase in viral replication was observed beginning at 12 h post‐infection in the 15°C and 20°C groups (Figure 1).

### 3.3 Comparison of Viral Adsorption Over Time Based on Exposure Duration

**FIGURE 2:**
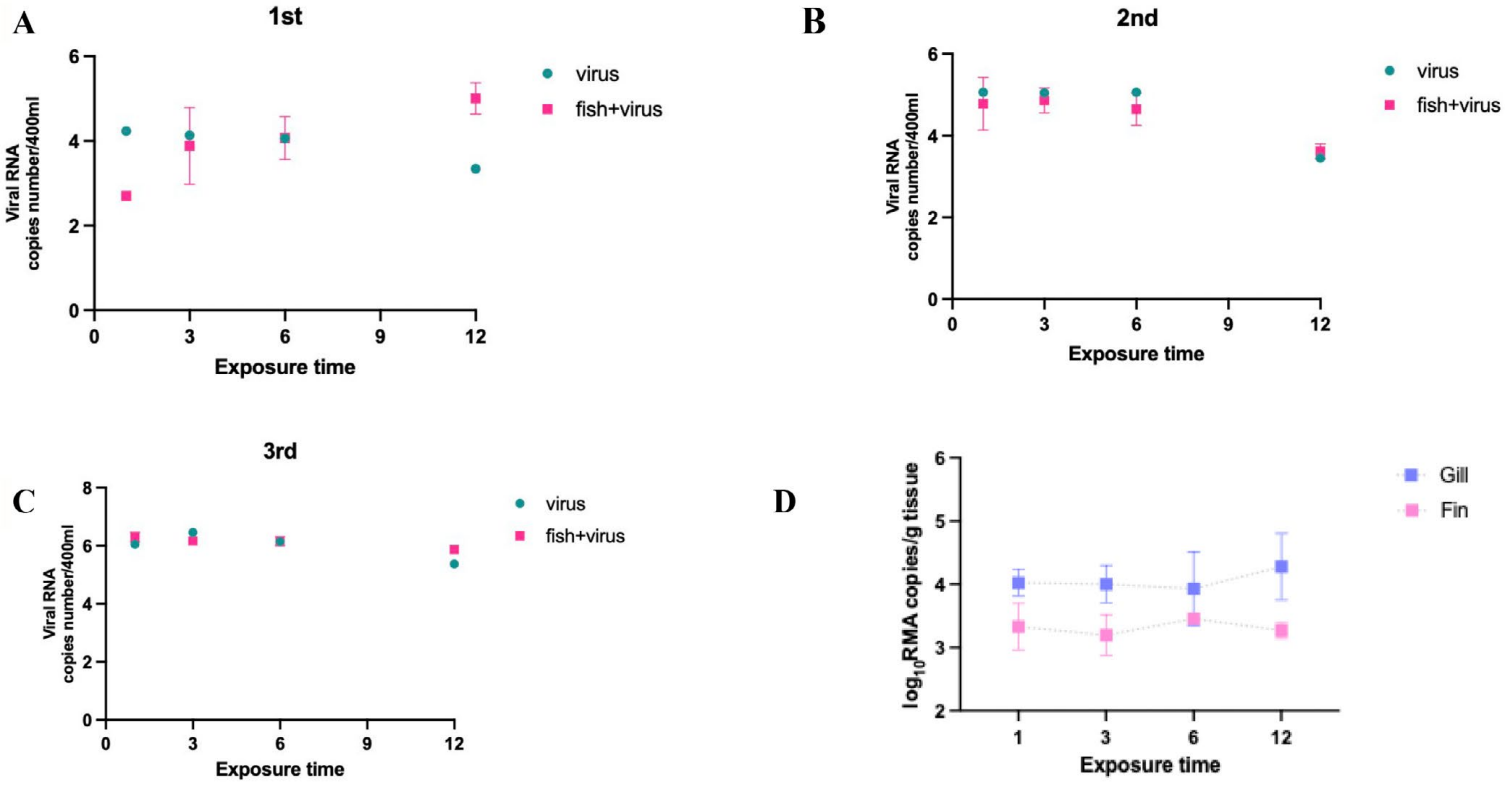
(A–C) VHSV adsorption kinetics in seawater were assessed under two conditions: seawater containing only the virus (virus only) and seawater containing both fish and virus (fish + virus) across three independent experiments (1st, 2nd, and 3rd). Viral RNA concentrations (copies/mL) were measured using qRT‐PCR at 0, 3, 6, 9, and 12 h post‐exposure. (D) Viral RNA levels in gill and fin tissues were quantified at the same time points to assess tissue‐specific VHSV uptake. Viral loads are expressed as log_10_ RNA copies per gram of tissue.

In the first experiment conducted at the initial aquaculture site, differences in viral load were observed between the virus‐only conditions and condition with fish at the 1‐h mark. However, this difference gradually decreased over time, suggesting that although the fish initially adsorbed the virus, it was likely re‐released back into the environment as time progressed (Figure 2).

In the second and third experiments at subsequent aquaculture sites, viral loads remained consistently high in the groups exposed only to the virus. From the 1–12 h post‐exposure, no significant difference in viral concentration was observed between the virus‐only condition and the condition with fish. This indicates that the fish at these sites either did not adsorb the virus or rapidly released it back into the water following initial adsorption. Furthermore, at the 12‐h mark in all experiments, viral loads were higher in the groups that included fish than in the virus‐only groups. This supports the hypothesis that the virus was temporarily adsorbed by the fish and subsequently released back into the surrounding environment over time.

### 3.4 Comparison of Viral Adsorption in Gills and Fins

This study compared the adsorption dynamics of VHSV in the gills and fins, analysing changes over exposure duration of 1, 3, 6, and 12 h. Overall, the gills exhibited higher levels of RNA replication (log RNA copies/g tissue) compared to the fins (Figure 2), suggesting that gills, due to their direct exposure to the external environment, may be more susceptible to VHSV. Both tissues showed an increasing trend in viral adsorption over time. Notably, the highest level of VHSV replication in the gills was observed at the 12‐h post‐infection, indicating a gradual accumulation of the virus. Although detectable levels of VHSV were also adsorbed by the fins, the viral loads were comparatively lower than those recorded in the gills.

## 4 Discussion

This study identified the adhesion sites of VHSV in olive flounder. Analysis of VHSV replication across different tissues under varying temperature conditions revealed significant differences depending on the tissue type and temperature. VHSV was detected within 1 h post‐infection in gill tissues and mucus, with viral RNA levels measured at 10^2^/μg, suggesting that these tissues serve as primary contact sites for viral adhesion. In the mucus, a marked increase in viral load was observed at 12 h post‐infection in the groups maintained at 15°C and 20°C. However, no significant viral replication was detected at 25°C, a temperature at which VHSV typically cannot survive. Gill tissues exhibited the highest viral loads between 1 and 3 h post‐infection, followed by a gradual decrease. However, in the 15°C group, viral replication persisted beyond 24 h. In contrast, systemic tissues, such as the spleen, kidney, muscle, and blood showed sustained viral replication beginning at 12 h post‐infection in both the 15°C and 20°C groups. These findings are consistent with previous studies (Jang et al. 2025), which demonstrated that VHSV replication is favoured at temperatures between 15°C and 20°C but is significantly inhibited at temperatures above 25°C. These results suggest that viral adhesion initially occurs in external tissues before the virus spreads to internal organs. Over time, the decrease in viral load within the gill tissues may result from the detachment of virus particles initially bound to the mucus as it is shed, or from the migration of the virus to target tissues, leading to a reduction in viral presence in the gills.

A comparative analysis of VHSV adsorption in gill and fin tissues over time revealed that the gills consistently exhibited a higher number of viral RNA copies across all exposure periods. This finding indicates that the gills are highly susceptible to VHSV attachment and entry, likely due to their extensive surface area and continuous exposure to waterborne pathogens. The epithelial structure and biochemical properties of the gills may contribute to increased viral binding, potentially through the presence of specific viral receptors or a more permeable epithelial barrier. In contrast, fin tissues exhibited relatively lower levels of viral adhesion.

Notably, the fin tissues maintained a consistently lower levels of viral attachment throughout the study, which contrasts with previous research by Montero et al. (2011), who reported higher VHSV replication in the fins compared to the gills of immersion‐infected rainbow trout. Additionally, studies employing recombinant VHSV proteins and bioluminescence imaging have predominantly detected viral presence in the fin rather than in the gill (Baillon et al. 2020). Despite these discrepancies, our findings support the hypothesis that the gills play a crucial role in the early stages of VHSV infection. Although our study did not assess chemokine gene expression (Baillon et al. 2020), previous studies have reported widespread activation of antiviral immune genes and chemokines in gill tissues following VHSV exposure, indicating an active immune response (Montero et al. 2011; Aquilino et al. 2014). Most prior studies have focused on detecting VHSV in host tissues after 24 h post‐infection, based on the typical onset of active viral replication, which occurs approximately 12 h post‐infection (Montero et al. 2011; Baillon et al. 2020). However, in our study, VHSV RNA copies were detected in gill tissues as early as 1–3 h post‐exposure, suggesting that the gills may serve as primary sites for viral adsorption during the early stages of infection. Furthermore, a sharp increase in viral RNA copies in the mucus layer was observed at 12 h post‐infection, which aligns with previous studies reporting viral replication in the fin tissues at 24 h post‐infection.

An important factors to consider is the potential role of gill mucus (Palaksha et al. 2008; Aquilino et al. 2014). Our findings indicate that VHSV RNA copies were detected in the gill tissue at an early stage of exposure, consist with results from previous experiments (Qadiri et al. 2020). These findings suggests that the gills may serve as an initial site for viral attachment prior to systemic dissemination. Aquilino et al. (2014) reported upregulation of the immune‐related genes IgM and IgT, which are predominantly associated with mucus, in gill tissues during early infection. This raises the question of whether the early detection of VHSV in the gills is attributable to viral attachment to epithelial cells or its presence within the mucus layer. Previous studies have also indicated that fish mucus can act as a viral reservoir, facilitating both viral adhesion and modulation of host immune responses (Salinas et al. 2011; Liang et al. 2022). Therefore, to accurately determine the exact site of viral attachment, further research using primary gill cell culture models is required to confirm cellular attachment and viral replication.

In this study, we also evaluated the efficiency of a seawater concentration method for the recovery and quantification of VHSV and compared VHSV adsorption in olive flounder. Using an Amicon Ultra‐15 centrifugal filter (30 kDa), we demonstrated high recovery rates, confirming the method's effectiveness for concentrating VHSV concentration from seawater samples. Furthermore, as the initial concentration of the viral inoculum increased, the recovery rate also improved. Notably, at higher viral loads, the recovery rate slightly exceeded 100% (Ryu et al. 2023). This observation aligns with findings from other studies, which suggest that ultrafiltration methods may occasionally overestimate recovery rates due to viral clustering or viral aggregate concentrations.

Using Centricon‐based concentration methods, we examined the adsorption dynamics of VHSV in an environment containing fish, compared to a virus‐only control. Following 1 h of exposure, viral loads in the fish‐containing tanks were lower than those in the virus‐only condition, indicating that VHSV rapidly adheres to host tissues upon initial contact. However, over time, the difference in viral concentrations between the two conditions decreased, suggesting that the virus may be gradually re‐released from host tissues into the surrounding water. In contrast, at the 12‐h time point, all three replicates of the virus‐only condition exhibited a decrease in viral RNA copy numbers, implying that VHSV may also adhere to tank surfaces or other environmental substrates (Joiner et al. 2021). Collectively, these findings suggest that both host‐associated and environmental factors may play a critical role in the environmental stability and persistence of VHSV within aquaculture systems.

Similar patterns have been observed in previous studies, where infected fish functioned as viral reservoirs, intermittently releasing viral particles into the surrounding water and thereby facilitating sustained transmission (Joiner et al. 2023). The dynamic equilibrium between viral attachment and shedding is particularly important in aquaculture environments, where high stocking densities can amplify the risk of viral transmission. The ability of VHSV to adhere to and subsequently detach from host tissues suggests that effective disease management strategies should not only focus on preventing initial viral exposure but also on controlling viral shedding and environmental contamination. Therefore, while our findings suggest that viral attachment and potential re‐release into the environment may contribute to the persistence of VHSV in aquaculture settings, further investigation is necessary to elucidate the mechanisms of viral shedding and environmental contamination. Such insights will be essential for developing comprehensive disease management strategies that mitigate both initial viral exposure and environmental persistence.

## Author Contributions

**Su‐Young Yoon:** writing – original draft, visualization, validation, methodology, formal analysis, data curation. **Yo‐Seb Jang:** investigation, formal analysis, software. **Soo‐Jin Kim:** methodology, conceptualization. **Myung‐Joo Oh:** writing – review and editing, supervision, resources, project administration, funding acquisition.

## Conflicts of Interest

The authors declare no Conflicts of Interest.

## Data Availability

The data that support the findings of this study are available on request from the corresponding author. The data are not publicly available due to privacy or ethical restrictions.
